# Production of Acid and Rennet-Coagulated Cheese Enriched by Olive (*Olea europaea* L.) Leaf Extract—Determining the Optimal Point of Supplementation and Its Effects on Curd Characteristics

**DOI:** 10.3390/foods13040616

**Published:** 2024-02-18

**Authors:** Elizabeta Zandona, Lucija Vranković, Sandra Pedisić, Tomislava Vukušić Pavičić, Ana Dobrinčić, Nives Marušić Radovčić, Katarina Lisak Jakopović, Marijana Blažić, Irena Barukčić Jurina

**Affiliations:** 1 Department of Food Technology, Karlovac University of Applied Sciences, Trg J.J. Strossmayera 9, 47000 Karlovac, Croatia; elizabeta.zandona@vuka.hr (E.Z.); marijana.blazic@vuka.hr (M.B.); 2 Department of Food Engineering, Faculty of Food Technology and Biotechnology, University of Zagreb, Pierottijeva 6, 10000 Zagreb, Croatiaspedisic@pbf.hr (S.P.); tomislava.vukusic.pavicic@pbf.unizg.hr (T.V.P.); adobrincic@pbf.hr (A.D.); nmarusic@pbf.hr (N.M.R.); klisak@pbf.unizg.hr (K.L.J.); 3 Gastronomy Department, Aspira University of Applied Sciences, Mike Tripala 6, 21000 Split, Croatia

**Keywords:** cheese, curd properties, olive leaf extract, phenols, flavonoids, antioxidant activity

## Abstract

This study investigated the potential of olive leaf extract (OLE), as a functional ingredient, to improve cheese properties, because it is rich in phenols. Milk and dairy products are poor in phenolic compounds. The main objective was to determine the most effective coagulation method and timing of OLE supplementation to maximize retention in the cheese matrix. Experimental cheeses were produced using the rennet and acid coagulation methods, with OLE added either directly to the cheese milk or to the curd phase. Three OLE effective concentrations corresponding to 25%, 50%, and 75% inhibition of DPPH reagent (EFC25, EFC50, and EFC75, respectively) were added, i.e., 11.5 mg GAE L^−1^, 16.6 mg GAE L^−1^, and 26.3 mg GAE L^−1^, respectively. The results showed that OLE significantly increased the concentration of total phenols, total flavonoids, and antioxidant activity in all cheese samples and in the residual whey, especially at higher effective concentrations (EFC 50 and EFC 75). Rennet-coagulated cheese to which OLE was added prior to coagulation (EM 25, EM 50, EM 75) exhibited higher hardness, gumminess, and chewiness but lower elasticity, suggesting alterations in the paracasein matrix. OLE did not adversely affect acidity, water activity, or cheese yield. However, higher EFC resulted in significant colour changes (∆E* > 3.0). In conclusion, the enrichment of cheesemaking milk with OLE and the application of the rennet coagulation method are the most suitable to optimise the production of OLE-enriched cheese. This research shows the potential to improve the nutritional value of cheese while maintaining its desired characteristics.

## 1. Introduction

The food industry is one of the largest producers of waste, such as leaves, peels, seeds, shells, pomace, etc., generated at the end of the food production process, which has led to great concern about its management and environmental footprint. According to the Food and Agriculture Organisation (FAO), the processing of plant foods, especially fruits and vegetables, results in the highest food losses along the food chain. Since the waste and/or byproducts generated very often contain components with antioxidant and antimicrobial properties (e.g., phenolic compounds), the food industry and food science have focused on exploring the possibilities of their use as novel ingredients for the production of functional foods [1,2]. In this context, plant byproducts are being extensively investigated for their reuse due to their potential health benefits and the fact that they are usually generated in the household before consumption and are thus readily available for further processing. Such an approach is favoured by modern consumers, who show a continued interest in healthier, clean-labelled, and natural foods. It also contributes to recent food sector priorities aimed at reducing food losses through the implementation of “zero waste” policies and sustainable food production systems [1,3,4,5]. Dairy products such as cheese and fermented milk are considered as some of the most popular functional food matrices due to their significant share in the dairy products market, frequent consumption, and acceptance by health-conscious consumers [6,7]. Moreover, cheese and fermented milk represent a suitable medium for fortification with nutritionally valuable compounds, especially plant extracts containing phenolic compounds that are absent in milk and dairy products [7,8,9]. Since Mediterranean agriculture relies heavily on the cultivation of olives and the production of olive oil [10], large quantities of olive leaves remain after processing [11]. Numerous previous studies have shown that olive leaf extract (OLE) is a rich source of various phenolic compounds with numerous health-protective properties (i.e., antioxidant, anticarcinogenic, anti-inflammatory, antihypertensive, cholesterol-lowering, etc.), of which oleuropein was found in the highest concentrations (up to 14%) [10,12,13,14,15,16]. The European Food Safety Agency (EFSA) has recognised the beneficial effects of OLE on human health, paving the way for the potential use of OLE to supplement foods to prevent various diseases and maintain human health [17]. Accordingly, several authors have explored the possibilities of adding OLE to dairy products [11,17,18,19]. In line with this, our previous study [8] focused on the supplementation of cow’s milk yoghurt with OLE. We found that the addition of OLE resulted in shorter fermentation and had no negative effects on the viability of yoghurt starter bacteria. OLE-fortified yoghurts had higher total phenolic content and antioxidant activity. Considering the above, the objective of this study was to determine which method of milk coagulation is most suitable to produce cheese enriched with OLE and at what point in the manufacturing process the addition of OLE is most appropriate to better retain the extract in the cheese. In addition, the influence of the coagulation method and the timing of the addition of OLE on the texture, colour, total phenolic and flavonoid content, and antioxidant activity of the cheeses produced was studied.

## 2. Materials and Methods

### 2.1. Olive Leaf Extract (OLE) Production and Composition

Olive leaves (*Olea Europaea* L., variety Oblica) harvested during the summer of 2021 in the Zadar County region (Croatia) were used for extract production. Leaves were air-dried after harvesting and milled prior to extraction. Subsequently, microwave-assisted extraction (MAE) was performed on Ethos X (Milestone, Bergamo, Italy), according to the previously optimized procedure by Dobrinčić et al. [10] with some modifications. Each extraction cell contained 6 g of milled olive leaves and 80 mL of distilled water as extraction solvent. Other extraction conditions were as follows: temperature (80 °C), pressure (100 bar), extraction time (2 min), time to reach the extraction temperature (2 min), mixing (50%), and cooling (1 min). The obtained OLE was filtered, pasteurized (5 min/90 °C), and cool stored at 4 °C until evaporation. The evaporation was performed on Buchi R-200 Rotavapor with Heating Bath B-490 (Büchi, Switzerland) until approximately 16% dry matter. The obtained concentrated OLE was freeze stored at −18 °C until further use. The composition of the produced OLE was determined by HPLC analysis with Agilent 1260 Infinity quaternary LC system (AgilentTechnologies, Santa Clara, CA, USA) equipped with photodiode array detector (PDA), an automatic injector, and ChemStation software (version C.01.03, Agilent Technologies, Santa Clara, CA, USA) which the solvent composition and the gradient conditions were described previously by Dragović-Uzelac et al. [20]. The phenolic compounds were identified by comparing the retention times and spectral data with those of the authentic standards, and quantification was performed by the external standard calculation, using the calibration curves of the authentic standards. The obtained OLE contained on average 1.70 mg g^−^^1^ oleuropein; 0.72 mg g^−^^1^ rutin; 0.38 mg g^−^^1^ tyrosol; 0.30 mg g^−^^1^ caffeic acid; and 0.26 mg g^−^^1^ chlorogenic acid, with the sum of total phenolics of 35.23 ± 0.79 mg GAE g^−^^1^, total flavonoids of 3.56 ± 0.14 mg QE g^−^^1^, and antioxidant activity of 38.67 ± 1.35 mg GAE L^−^^1^ obtained by using the DPPH method [21].

#### Determination of OLE Effective Concentrations

OLE concentrations used in the production of cheeses were determined considering the antioxidant activity by using the DPPH method according to Tavakoli et al. [21]. After determining the antioxidant activity of the prepared OLE solutions of known concentrations, an inhibition curve was established from the DPPH reagent inhibition values by using the GraphPad Prism version 8.3.0. (GraphPad Software, La Jolla, CA). The obtained inhibition curve was further used to determine the effective concentrations of OLE that inhibit 25, 50, and 75% of the DPPH reagent (EFC 25, EFC 50, and EFC 75), which were 11.5; 16.6; and 26.3 mg GAE L^−^^1^, respectively.

### 2.2. Production of Cheese with OLE Addition

**Figure 1:**
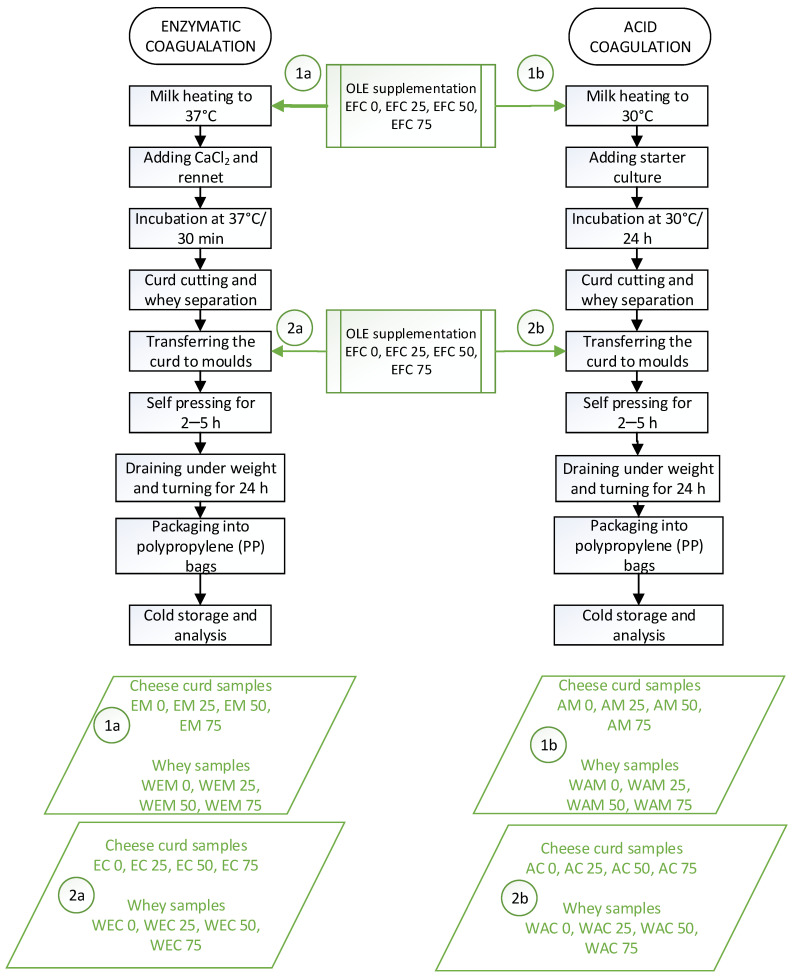
The scheme of cheese curd production enriched with olive leaf extract (OLE) at different stages of production.

The cheese curds were produced on a lab scale from commercially available pasteurised, non-homogenized milk with 3.2% milk fat, 4.8% carbohydrates, 3.3% proteins (Veronika mini dairy, Desinić, Croatia) by enzymatic (sample codes EM and EC) and acid (samples code AM and AC) coagulation, respectively (Figure 1). In the experimental setup, 4 L of milk was utilized for the production of each cheese curd sample. For each coagulation method, two groups of cheeses were produced depending on the moment of OLE addition, whereby three different effective concentrations of OLE (EFC 25, EFC 50, and EFC 75) were applied. For each coagulation method, OLE was added either directly into the milk before coagulation or into the obtained curd. Depending on the coagulation method, control cheeses without OLE (EFC 0) were produced as well. Also, whey samples remaining after each production process (sample codes: WEM and WEC for enzymatic coagulation; WAM and WAC for acid coagulation) were collected and cool stored until further analysis (Figure 1). For cheeses produced by enzymatic coagulation, calcium chloride (CaCl_2_) (Gram-mol, Croatia) and natural rennet (IMCU 1170, chymosin 90%, pepsin 10%) (Caglificio Clerici, SIRIS, Lub do.o., Split, Croatia) were added according to the manufacturer’s instructions. The acid coagulation method included inoculation by adding 10 DCU of starter cheese culture (LYO 100 DCU, Danisco, Denmark). All the cheese curds were pressed by their own weight for approximately 2–4 h and then subjected to a 5 kg weight for 24 h with occasional turning. After removing the cheeses from the mould, they were weighed and packed in hermetically sealed, disinfected polypropylene bags at the end of the production process and cool stored at +4 °C until further analysis.

### 2.3. Physicochemical Composition and Water Activity Measurements

Physicochemical composition of curds (moisture, total solids, fat, protein, fat in dry matter—FDM, and saturated fatty acids—SFA) was determined using NIR (near infrared) transmission technology by FoodScan™ 2 Lab with FOSS ANN Dairy Calibration (FOSS Analytical A/S). Measurements were conducted on 15 g of crushed curd in a plastic Petri dish (diameter 90 mm). Active acidity (pH) of cheese was determined by using a pH meter ProfiLine pH 3110 (Xylem Analytics, Weilheim in Oberbayern, Germany) according to Božanić et al. [22]. pH value of whey samples was also determined, without prior sample preparation. Titratable acidity (°SH) of cheese samples was determined according to the AOAC standard method [23]. Water activity (a_w_) of cheese was determined by using a HygroPalm HP23 a_w_ meter (Rotronic, Bassersdorf, Switzerland).

### 2.4. Colour Determination

The colour of cheese curd samples was determined according to the CIElab system using a CM-CM-700d colorimeter (Konica Minolta, Tokyo, Japan) with D65 light source, as previously described in the study by Barukčić et al. [8]. L*, a* and b* parameters were measured and the total colour difference (ΔE*) was calculated following Equation (1):

(1) $${∆E}^{*}=\sqrt{{\left(L^{*}-L_{ref}^{*}\right)}^{2}+{\left(a^{*}-a_{ref}^{*}\right)}^{2}+{\left(b^{*}-b_{ref}^{*}\right)}^{2}},$$

where L*, a*, and b* refer to the test samples and $L_{ref}^{*}$, $a_{ref}^{*}$, and $b_{ref}^{*}$ to the control samples. The obtained results were interpreted according to the model described by Mokrzycki and Tatol [24] (Table 1).

### 2.5. Texture Determination

The texture of cheese curds was determined using a texture analyser (Ametek Lloyd Instruments Ltd., West Sussex, UK) as Marušić Radovčić et al. [25] previously described. Cheese samples were twice compressed up to 50% of deformation with a 50 kg cell, at a speed of 1 m s^−^^1^ with a 5-s interval between two cycles. The obtained data were processed using NexygenPlus software version 3.0 (Ametek Lloyd Instrument LTD, West Sussex, UK) hich generated the values for adhesiveness (Nmm), adhesive force (N*), cohesiveness, hardness (N), gumminess (N), chewiness (Nmm), stringiness (mm), resilience, fracture, (N) and springiness (mm).

* N standing for newton (The SI unit of force)

### 2.6. Spectrophotometric Analyses of Cheese Curds and Whey Samples

#### 2.6.1. Sample Preparation

The OLE and whey samples were diluted with distilled water to achieve acceptable absorbance results (between 0 and 1). Cheese curd samples were prepared according to Apostolidis et al. [26] by homogenising 20 g of cheese with 20 mL of distilled water and centrifuging the prepared suspension at 10,000 rpm for 10 min at 5 °C. The obtained supernatant was separated and stored at 4 °C until further analysis.

#### 2.6.2. Total Phenol Content

The total phenol content was determined by using the Folin–Ciocalteu method, modified according to Shortle et al. [27]. The standard curve was plotted using 500 mg L^−^^1^ gallic acid (Sigma-Aldrich, St. Louis, MO, USA) as a stock solution, and Equation (2) was obtained as follows:

(2) $$Y=0.0031\times X\;(R^{2}=0.9947),$$

#### 2.6.3. Total Flavonoid Content

The total flavonoid content was determined according to Aryal et al. [28] with slight modifications. The standard curve was derived using 2 mmol L^−^^1^ quercetin (Sigma-Aldrich, St. Louis, MO, USA) as a stock solution, from which Equation (3) was obtained:

(3) $$Y=0.0098\times X\;(R^{2}=0.9981),$$

#### 2.6.4. Antioxidant Activity by Using the FRAP Method

The antioxidant activity of cheese and whey samples was determined by using the ferric reducing antioxidant power (FRAP) method according to Benzie et al. [29]. The standard curve was plotted using 1 mmol L^−1^ Trolox (Sigma-Aldrich, St. Louis, MO, USA) as a stock solution, from which Equation (4) was obtained:

(4) $$Y=0.0014\times X\;(R^{2}=0.9996)$$

### 2.7. Statistical Analysis

Each experiment was repeated in triplicate, and the obtained results were expressed as mean values ± standard deviations (SDs). Three-way analysis of variance (ANOVA) was performed to determine the significant effect of factors as well as their interactions on the examined physicochemical and textural parameters, total phenols, flavonoids, and antioxidant activity. In this regard, one factor was the method of cheese milk coagulation (enzyme or acid), another factor was the moment of OLE addition (prior to coagulation or curd supplementation), and the third factor was the effective concentration of added OLE (EFC 25, EFC 50, and EFC 75). Multiple comparison of means within groups was performed with Tukey’s HSD test using GraphPad Prism software. The significance level for all tests was set at *p* ≤ 0.05.

## 3. Results and Discussion

### 3.1. Physicochemical Composition of the Produced Cheese Curds

The physicochemical composition of the cheese curds is presented in Table 2. The effective concentration of OLE and the coagulation method had a significant effect on moisture, fat, protein, SFA, FDM, and total solids (*p* < 0.0001). Moisture content ranged from 67.385 ± 1.22 (EC 75) to 79.88 ± 0.66% (AM 0), with higher values for acid-coagulated cheese curds, which are in accordance with those previously reported for soft cheese [30]. Furthermore, acid-coagulated cheese curds had a lower fat, protein, SFA, and FDM content than rennet-coagulated cheese curds. The moisture content for rennet-coagulated curds was somewhat higher than usually reported for this type of coagulation [30,31,32]. Such results might be explained by the fact that this study focused on drained cheese curds without additional treatments such as salting or aging, which usually reduce the moisture content in cheese.

Since the water activity (a_w_) of cheese curds supplemented with OLE before coagulation did not increase compared to the control sample, the increase in total solids and protein content is most likely due to the proposed casein-polyphenol complexes. Due to protein dissociation and changes in ionic strength that occur naturally at lower pH values, the acidification of milk that occurs during fermentation increases the number of binding sites on proteins. Accordingly, the observed higher total solids, protein content, and a_w_ values (Table 2) indicate that the added OLE polyphenols most likely formed complexes with milk proteins (especially caseins), which was more intense in samples supplemented with higher effective concentrations before coagulation. These observations could be additionally confirmed by the results of colour measurement (Table 2), as the effective concentration and the time of OLE addition showed a statistically significant (*p* < 0.01) influence on the measured values of the parameters L*, a*, and b* and, accordingly, on the calculated ΔE* values of the supplemented cheeses, regardless of the coagulation method (*p* > 0.05). More specifically, ΔE* values were significantly (*p* < 0.0001) higher in cheeses supplemented with OLE before coagulation than in control cheeses and cheeses supplemented after coagulation (quark supplementation). Thereby, the ΔE* values increased with the increase of the effective OLE concentration in both types of supplements and coagulation methods. However, the obtained ΔE* values of enzyme-coagulated cheese curds were significantly lower than those of acid-coagulated cheese supplemented at the same time of the production process (Table 2). All cheeses supplemented prior to coagulation had ΔE* values greater than 3.0, indicating colour changes visible to the human eye [24,37]. These results indicate that acid coagulation led to more intense interactions between the added OLE components and the cheese proteins, and thus to the preservation of the bioactive components of OLE in the produced cheeses. Accordingly, these cheese samples were characterised by more intense colour changes. Barukčić et al. [8] previously found similar observations in fermented milk supplemented with OLE, as did Roseiro et al. [38], who studied the mechanisms of the binding of polyphenols to milk proteins.

### 3.2. Textural Parameters of OLE Supplemented Cheese Curds

**Figure 2:**
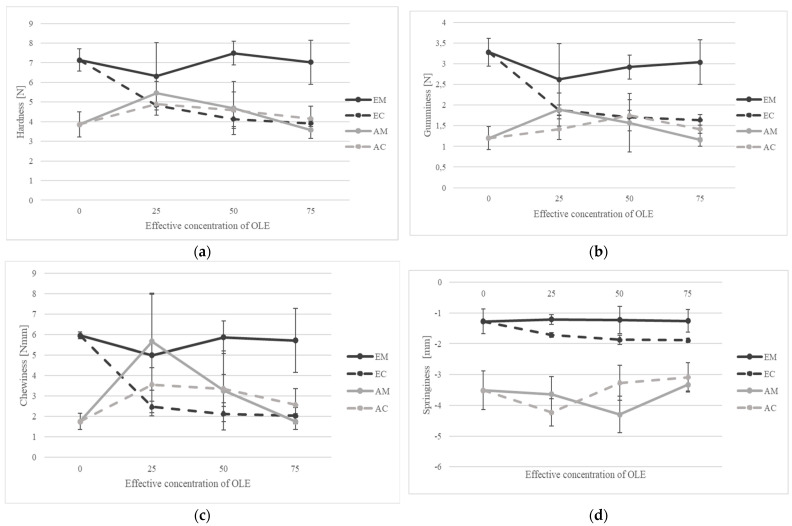
The textural properties of (**a**) hardness, (**b**) gumminess, (**c**) chewiness, (**d**) and springiness of OLE-enriched enzymatic (EM, EC) and acid-coagulated (AM, AC) cheese curds in relation to the added effective olive leaf extract (OLE) concentrations (0, 25, 50, 75). The addition of OLE to milk is represented by a solid line, while the dashed line corresponds to the addition of OLE to cheese curds. The black lines indicate enzymatic coagulation, while the grey ones correspond to the acid coagulation.

Statistically significant differences (*p* < 0.05) between samples were found for hardness (N) (Figure 2a), gumminess (N) (Figure 2b), chewiness (Nmm) (Figure 2c), and springiness (mm) (Figure 2d). Cheese hardness is quantified as the force required to cause the deformation of the cheese, while gumminess is defined as the energy required to crush the cheese to the point where it can be swallowed. Elasticity refers to the ability of the cheese to regain its original shape after initial compression, while chewiness represents the energy required to chew the cheese to a consistency suitable for swallowing.

The authors observed significantly lower values of hardness in all examined cheese curds, with values ranging from 3.57 ± 0.43 (AM 75) to 7.49 ± 0.61 N (EM 50). Such low values of hardness are not expected for traditionally produced rennet-coagulated cheese, but these results may be related to the high retention of moisture in cheese curds supplemented with high levels of OLE. When comparing between the results of the physicochemical composition (Table 2) and hardness of cheese curds (Figure 2a), it becomes clear that the curd hardness highly depends on moisture, fat, and protein content. Cheese curds with OLE supplemented to milk prior to coagulation achieved significantly (*p* < 0.0001) higher hardness, gumminess, and chewiness than cheese curds with OLE supplementation after the coagulation, regardless of the coagulation method. A drop in temperature during curd supplementation with OLE apparently resulted in the weaker binding of cheese granules and affected the texture parameters in curd-supplemented cheeses. Enzyme-coagulated cheese curds supplemented with OLE prior to coagulation (EM 25, EM 50, EM 75) achieved the highest hardness, gumminess, and chewiness and the lowest brittleness among all enzyme-coagulated cheese curds and among all manufactured cheese curds, regardless of the coagulation method. These results indicate a change in the paracasein matrix and are consistent with previous studies [39]. The binding of OLE polyphenols to casein appeared to strengthen the curd network and increase cheese curd firmness. Cheese composition also affects the final texture, as both dissolved calcium in the cheese serum and calcium bound to the protein network have been shown to affect the rate of proteolysis, and cheeses having a higher fat content are less firm and more elastic [40]. It is important to highlight that, particularly in the context of enzymatic coagulation, the results refer to drained curds before the salting stage, as this differentiation exerts a substantial influence on the texture parameters. To better understand the matrix changes, future studies should provide a detailed analysis of the final cheese composition in relation to the textural parameters. In general, it was observed that cheese curds enriched with OLE produced by acid coagulation had significantly lower hardness (*p* < 0.0001), gumminess (*p* < 0.0010), and chewiness (*p* < 0.0100), and higher brittleness (*p* < 0.0010), regardless of cheese milk fortification or cheese curd supplementation, again suggesting a change in the protein network. As mentioned by Lucey et al. [41], acidity plays an important role in determining the textural properties of cheese, a finding consistent with the results of our study. In our study, we observed slightly higher pH values when OLE was added directly to milk compared to other methods. Table 2 shows that the samples to which OLE was added directly at a concentration equivalent to EFC 50 had the highest titratable acidity values. This indicates that the addition of OLE to the milk affected the acidity of the cheese and likely contributed to the observed changes in texture. The results of the three-way ANOVA confirmed a significant difference between the coagulation methods and the time points of OLE supplementation in terms of hardness (*p* < 0.0001), gumminess (*p* < 0.0001), chewiness (*p* = 0.0002), and elasticity (*p* = 0.0255), regardless of the effective OLE concentration (*p* > 0.05) (Table S2).

### 3.3. Total Phenols, Flavonoids, and Antioxidant Activity of the Produced Cheese Curds and Remaining Whey

#### 3.3.1. Total Phenols (TP)

**Figure 3:**
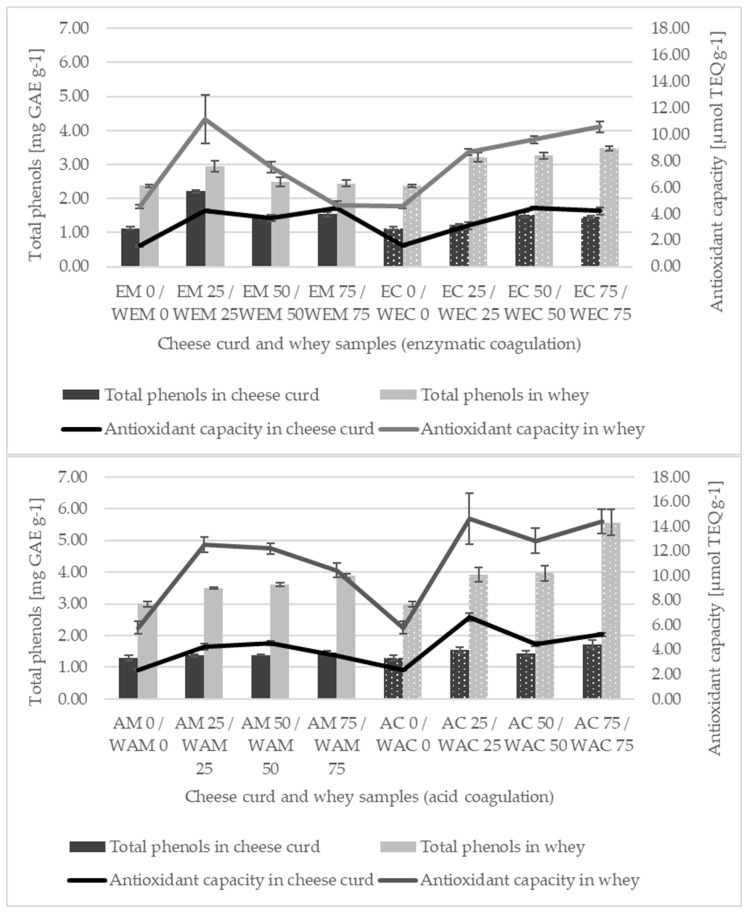
Total phenols (TP) (mg GAE g^−1^) and antioxidant activity (AA) (µmol TEQ g^−1^) in olive leaf extract (OLE) (0, 25, 50, 75) enriched cheese curds produced by rennet (EM, EC) (**a**) or acid coagulation (AM, AC) (**b**) and the remaining whey (WEM, WEC, WAM, WAC). Black columns indicate TP in cheese curd samples, while grey columns correspond to TP in whey samples. Solid fill represents milk supplementation with OLE, while the dotted pattern indicates curd supplementation with OLE. The black line represents an AA (µmol TEQ g^−1^) in cheese curd samples, while the grey line corresponds to AA in whey samples.

The total phenolic concentration (TP) varied between 1.13 ± 0.04 (EM 0) and 2.21 ± 0.03 mg GAE g^−^^1^ (EM 25) for enzymatic coagulation (Figure 3a) and between 1.29 ± 0.08 mg GAE g^−^^1^ (AM 0) and 1.73 ± 0.14 mg GAE g^−^^1^ (AC 75) for acid coagulation (Figure 3b), respectively. The TP in the remaining whey varied between 2.37 ± 0.04 (WEM 0) and 3.48 ± 0.06 mg GAE g^−^^1^ (WEC 75) for enzymatic coagulation and between 2.98 ± 0.09 (WAM 0) and 5.57 ± 0.40 mg GAE g^−^^1^ (WAC 75) for acid coagulation, respectively. In general, it was found that the addition of OLE significantly (*p* < 0.0001) increased the TP in all cheese samples as well as in the remaining whey, regardless of the coagulation method (*p* > 0.05). The obtained results are consistent with previously published data [8,42,43,44,45]. The increase in TP corresponds to the increase in the effective concentration of the added OLE, except in the sample EM 25, where the TP was the highest, probably due to the variations in the dewatering process. The difference between the effective concentrations and the times of the addition of OLE was significant (*p* < 0.0001). Due to the low molecular weight and good solubility properties of phenolic compounds, the production of cheese from OLE-fortified milk resulted in the better retention of phenols in cheese compared to supplementation with curds. The retention of phenolic compounds in cheese is due to hydrophobic, hydrophilic, ionic, or covalent interactions between phenolic compounds and milk proteins [46]. The mechanism of these interactions is not yet fully understood, and further studies to increase the retention coefficient and the possibility of producing functional products are of interest [47]. Indeed, soluble compounds of low molecular weight are often lost to a great extent in cheese whey. The increased levels of the TP in all samples of collected whey may indicate limited retention in the cheese matrix. Antioxidative activity in whey is primarily dependent on whey proteins and their peptides [48], formed by enzymatic breakdown, which could explain the observed results. Furthermore, whey proteins exhibit higher antioxidative activity than caseins, and previous studies have demonstrated that certain milk components, such as B-group vitamins, proteins, amino acids, etc. [49], can participate in reactions with the Folin–Ciocalteu reagent. Moreover, a positive correlation between TP determination with the Folin–Ciocalteu reagent and FRAP in milk has been documented [50], and this should be taken into consideration when interpreting the results of the TP in the collected whey samples.

#### 3.3.2. Total Flavonoids (TF)

**Figure 4:**
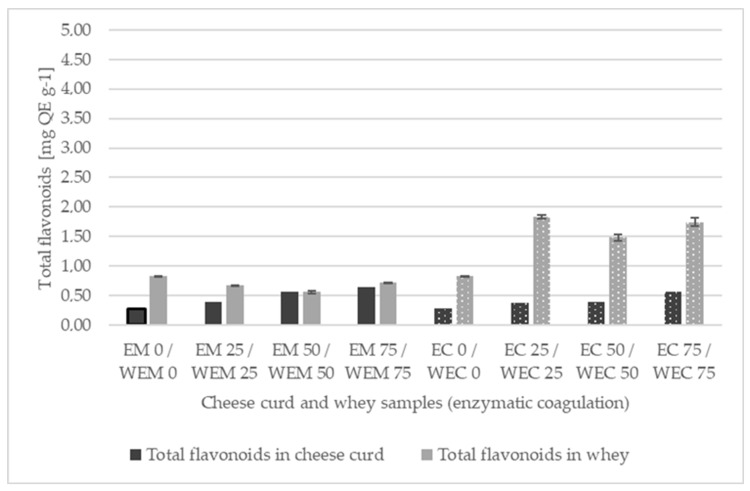

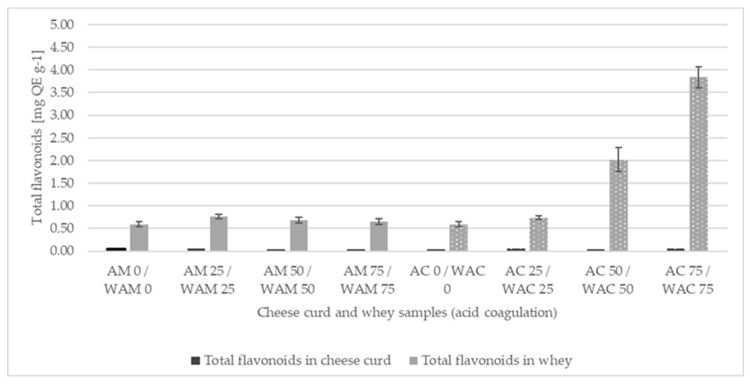
Total flavonoids (mg QE g^−1^) in olive leaf extract (OLE) (0, 25, 50, 75) enriched cheese curds produced by rennet (EM, EC) (**a**) or acid coagulation (AM, AC) (**b**) and the remaining whey (WEM, WEC, WAM, WAC). Black columns indicate TF in cheese curd samples, while grey columns correspond to TF in whey samples. Solid fill represents milk supplementation with OLE, while the dotted pattern indicates curd supplementation with OLE.

The concentration of total flavonoids (TF) in cheese varied between 0.28 ± 0.02 (EM 0) and 0.65 ± 0.01 mg QE g^−^^1^ (EM 75) in enzymatic coagulation (Figure 4a) and between 0.04 ± 0.01 (AC 50) and 0.06 ± 0.01 mg QE g^−^^1^ (AC 75) in acid coagulation (Figure 4b), respectively. The TF in the remaining whey varied between 0.65 ± 0.03 (WEM 50) and 0.82 ± 0.01 mg QE g^−^^1^ (WEM 0) for enzymatic coagulation and between 0.59 ± 0.06 (WAM 0, WAC 0) and 3.84 ± 0.23 mg QE g^−^^1^ (WAC 75) for acid coagulation, respectively. According to the three-way ANOVA, all three factors (coagulation method, time of supplementation, and effective concentration) had a significant effect (*p* < 0.0001) on TF in cheese. Enzyme coagulation resulted in significantly (*p* < 0.0001) better retention of flavonoids in cheese compared to acid coagulation, regardless of whether OLE was added to the milk or curds. Interestingly, even the control samples (AM 0, AC 0) had remarkably low TF values, which then increased slightly with the addition of OLE. The TF values obtained are similar to those obtained by Frühbauerová et al. [51] when processed cheese was fortified with oven-dried and freeze-dried grape skin powder. However, taking into account some previous studies, the interactions between flavonoid structures and milk proteins become more stable when the pH is closer to the isoelectric point of the protein [38], and the above pH values (Table 2) of acid-coagulated cheeses are close to this point, so the formation of a complex between casein and flavonoids could complicate the analysis, thus the influence of the coagulation method on TF values cannot be clearly highlighted. In general, milk fortification resulted in the better retention of flavonoids than the addition of curds, for both enzyme-coagulated and acid-coagulated cheeses. This is also confirmed by the TF values for the residual whey (Figure 4), which were significantly higher for WEC 25, WEC 50, WEC 75, WAC 25, WAC 50, and WAC 75 compared to WEM 25, WEM 50, WEM 75, WAM 25, WAM 50, and WAM 75. Increasing the effective concentration of OLE resulted in a linear increase in TF values regardless of whether OLE was added to the milk or curd prior to enzyme coagulation, whereas this was not the case for acid-coagulated cheeses.

#### 3.3.3. Antioxidant Activity (AA)

The antioxidant activity (AA) of cheese ranged from 1.58 ± 0.06 (EM 0) to 4.45 ± 0.14 µmol g^−^^1^ (EM 75) for enzymatic coagulation (Figure 3a) and from 2.37 ± 0.05 (AM 0) to 6.63 ± 0.34 µmol g^−^^1^ (AC 25) for acid coagulation (Figure 3b), respectively. The AA of the remaining whey ranged from 4.55 ± 0.12 (WEM 0, WEC 0) to 11.14 ± 1.83 µmol g^−^^1^ (WEM 25) for enzymatic coagulation and from 5.79 ± 0.51 (WAM 0, WAC 0) to 14.63 ± 2.08 µmol g^−^^1^ (WAC 25) for acid coagulation, respectively. When comparing the AA values of control cheese (EM 0, EC 0, AM 0, AC 0) and OLE-enriched cheese (Figure 3a,b), it was found that OLE enrichment significantly (*p* < 0.0001) increased the AA value, regardless of the coagulation method and the time of OLE enrichment. The same trend was also observed for TP, which is in agreement with [52], who found a positive correlation between AA and total polyphenol content. Numerous studies have already shown that the AA of dairy products can be significantly improved by adding sources of various phytochemicals [9,44,53]. According to the three-way ANOVA, all three factors (coagulation method, time of supplementation, and effective concentration) had a significant effect (*p* < 0.0001) on the AA in cheese. The highest AA was observed in acid-coagulated cheese curds to which OLE was added directly to the curds. An increase in AA was also observed in the whey remaining after acid coagulation, which was significantly higher compared to enzymatic coagulation (*p* < 0.0001), correlating with the results of the TP (Figure 3). Although the influence of OLE on the AA of cheese has not been studied in detail, some previous studies have also shown that the addition of OLE leads to an increase in the antioxidant activity of yogurt [8,19] and kefir [54]—dairy products that are also produced by acid coagulation.

## 4. Conclusions

The results of this study demonstrated that higher effective concentrations of OLE (EFC 50 and EFC 75) supplemented to rennet-coagulated cheese curds increased fat, protein, SFA, and total solids in cheese curds, likely due to polyphenol-casein complex formation, with notable colour changes. Acid-coagulated cheeses exhibited more pronounced colour changes compared to rennet-coagulated ones. Rennet-coagulated cheese curds supplemented with OLE before coagulation (EM 25, EM 50, EM 75) displayed elevated hardness, gumminess, and chewiness but reduced elasticity, indicating paracasein matrix changes. OLE significantly increased the total phenolic concentration, particularly at higher effective concentrations (EFC 50 and EFC 75). Cheese curds made from OLE-fortified milk exhibited better phenol retention compared to post-coagulation OLE supplementation. While the exact interaction mechanism between phenolic compounds and milk proteins requires further exploration, these results suggest OLE’s potential to enhance cheese’s antioxidant and health-promoting properties, especially when added before rennet coagulation. However, further studies are required to understand the effects on cheese properties during the ripening process and shelf life.

## Figures and Tables

**Table 1:** Typical scale of the total colour difference for understanding human visual perception.

ΔE* Value	Observer Colour Difference Perception
0–1.0	Observer does not notice the difference
1.1–2.0	Only experienced observer can perceive the difference
2.1–3.5	Inexperienced observer also notices the colour difference
3.6–5.0	A clear distinction in colour is visible
>5.0	Observer notices two different colours

**Table 2:** Influence of the coagulation method (enzymatic/E or acid/A), effective concentration of olive leaf extract (OLE) (EFC 0, EFC 25, EFC 50, EFC 75), and the moment of OLE addition (milk/M or curd/C) on the physicochemical characteristics of OLE-enriched cheese curds.

Samples	pH	Acidity (% Lactic Acid)	a_w_	Fat (%)	Moisture (%)	Protein (%)	SFA ** (%)	FDM *** (%)	Total Solids (%)	ΔE*
	*p* = 0.0087 *	*p* < 0.0001 *	*p* = 0.0162 *	*p* = 0.0312 *	*p* = 0.0476 *	*p* = 0.0070 *	*p* = 0.1324	*p* = 0.0012 *	*p* = 0.0476 *	*p* < 0.0001 *
EM 0	6.95 ± 0.06 ^b^	0.33 ± 0.00 ^mn^	0.90 ± 0.01 ^abc^	9.97 ± 0.18 ^a^	74.49 ± 0.33 ^a^	9.50 ±0.12 ^a^	5.77 ± 0.18 ^a^	39.05 ± 0.21 ^a^	25.51 ± 0.33 ^a^	/
EM 25	6.83 ± 0.03 ^c^	0.21 ± 0.00 ^o^	0.91 ± 0.01 ^a^	12.21 ± 0.89 ^abc^	70.63 ± 1.40 ^a^	11.02 ± 0.49 ^abc^	7.63 ± 0.48 ^bcde^	41.55 ± 1.06 ^a^	29.37 ± 1.40 ^ab^	3.08 ± 1.04 ^b^
EM 50	6.87 ± 0.01 ^bc^	0.47 ± 0.00 ^hi^	0.92 ± 0.03 ^ab^	12.36 ± 1.15 ^abc^	70.58 ± 1.94 ^abcd^	10.72 ± 1.02 ^abc^	7.96 ± 0.59 ^bcde^	41.95 ± 1.20 ^a^	29.42 ± 1.94 ^ab^	3.31 ± 2.05 ^b^
EM 75	6.86 ± 0.03 ^bc^	0.42 ± 0.01 ^ijkl^	0.91 ± 0.00 ^ab^	11.13 ± 1.48 ^abc^	72.415 ± 2.62 ^ab^	10.19 ± 1.33 ^ab^	7.47 ± 0.42 ^bcde^	40.25 ± 1.48 ^a^	27.59 ± 2.62 ^ab^	3.46 ± 0.44 ^b^
EC 0	7.18 ± 0.03 ^a^	0.43 ± 0.01 ^hijkl^	0.94 ± 0.03 ^ab^	10.09 ± 0.37 ^a^	74.185 ± 1.07 ^a^	9.59 ± 0.38 ^a^	5.84 ± 0.27 ^a^	39.05 ± 0.21 ^a^	25.82 ± 1.07 ^a^	/
EC 25	7.19 ± 0.00 ^a^	0.39 ± 0.00 ^ijlm^	0.95 ± 0.01 ^abc^	12.81 ± 0.34 ^bc^	69.26 ± 0.83 ^b^	11.86 ± 0.28 ^bc^	7.98 ± 0.00 ^bcde^	41.65 ± 0.07 ^a^	30.74 ± 0.83 ^b^	1.27 ± 1.13 ^bc^
EC 50	7.21 ± 0.01 ^a^	0.40 ± 0.00 ^ijklm^	0.94 ± 0.02 ^a^	12.08 ± 0.29 ^abc^	70.725 ± 0.57 ^abcd^	11.04 ± 0.22 ^abc^	7.21 ± 0.04 ^bcd^	41.25 ± 0.21 ^a^	29.28 ± 0.57 ^ab^	1.55 ± 0.19 ^bd^
EC 75	7.23 ± 0.02 ^a^	0.37 ± 0.01 ^klmn^	0.95 ± 0.00 ^ab^	13.59 ± 0.56 ^bc^	67.385 ± 1.22 ^b^	12.70 ± 0.56 ^bc^	8.67 ± 0.39 ^bcde^	41.7 ± 0.14 ^a^	32.62 ± 1.22 ^b^	2.28 ± 0.35 ^c^
AM 0	5.11 ± 0.01 ^de^	1.57 ± 0.01 ^d^	0.93 ± 0.04 ^ab^	6.42 ± 0.23 ^d^	79.875 ± 0.66 ^c^	6.79 ± 0.19 ^de^	5.60 ± 0.09 ^a^	31.85 ± 0.07 ^bde^	20.13 ± 0.66 ^c^	/
AM 25	5.06 ± 0.02 ^d^	2.23 ± 0.03 ^a^	0.95 ± 0.01 ^ab^	6.71 ± 0.53 ^d^	78.025 ± 1.51 ^acd^	8.275 ± 0.53 ^ade^	5.62 ± 0.72 ^a^	30.5 ± 0.28 ^bde^	21.98 ± 1.51 ^acd^	5.59 ± 0.33 ^b^
AM 50	5.09 ± 0.01 ^de^	2.28 ± 0.01 ^b^	0.95 ± 0.00 ^ab^	6.85 ± 0.18 ^d^	77.07 ± 0.13 ^acd^	8.57 ± 0.21 ^ade^	6.48 ± 0.52 ^ab^	29.85 ± 0.64 ^bd^	22.93 ± 0.13 ^acd^	5.91 ± 0.16 ^b^
AM 75	5.03 ± 0.03 ^d^	1.61 ± 0.01 ^c^	0.96 ± 0.01 ^c^	6.89 ± 0.46 ^d^	77.01 ± 0.34 ^acd^	9.155 ± 0.04 ^a^	5.41 ± 0.12 ^a^	29.9 ± 1.56 ^bde^	22.99 ± 0.34 ^acd^	11.98 ± 1.18 ^a^
AC 0	4.91 ± 0.02 ^f^	1.43 ± 0.00 ^e^	0.96 ± 0.00 ^b^	6.41 ± 0.24 ^d^	79.87 ± 0.72 ^dc^	6.74 ± 0.21 ^de^	5.42 ± 0.12 ^a^	31.85 ± 0.07 ^b^	20.13 ± 0.72 ^cd^	/
AC 25	5.04 ± 0.01 ^d^	2.23 ± 0.00 ^a^	0.95 ± 0.01 ^ab^	9.42 ± 0.11 ^a^	74.705 ± 0.06 ^a^	8.99 ± 0.06 ^a^	5.86 ± 0.16 ^a^	37.25 ± 0.35 ^ac^	25.30 ± 0.06 ^a^	1.96 ± 0.85 ^b^
AC 50	5.12 ± 0.03 ^de^	1.34 ± 0.02 ^f^	0.95 ± 0.00 ^ab^	8.84 ± 0.42 ^ad^	74.98 ± 0.91 ^a^	8.44 ± 0.42 ^ade^	7.07 ± 0.13 ^abc^	35.3 ± 0.42 ^c^	25.02 ± 0.91 ^a^	2.49 ± 1.52 ^b^
AC 75	5.16 ± 0.02 ^e^	1.26 ± 0.00 ^g^	0.95 ± 0.01 ^ab^	8.16 ± 0.51 ^ad^	76.16 ± 0.88 ^acd^	7.89 ± 0.34 ^ade^	6.96 ± 0.21 ^abcd^	34.2 ± 0.85 ^bc^	23.84 ± 0.88 ^acd^	3.63 ± 0.44 ^b^

* *p* < 0.05. Results are expressed as means ± SD. Values with different exponential expressions within one column are statistically significant at *p* ≤ 0.05. ** SFA (saturated fatty acids). *** FDM (fat in dry matter). Another parameter that has been monitored is water activity (a_w_), which directly affects microbiological and enzymatic activity in products and significantly influences the quality of cheeses, especially hard cheeses, since numerous enzymatic reactions occur during the ripening process, resulting in specific sensory characteristics [33]. According to the results presented in Table 2, there was no significant difference in the values obtained between cheese curds with and without the addition of OLE, even with respect to the coagulation methods used. The results are in agreement with those obtained by Kaya and Öner [33] for semi-hard traditional Turkish cheese and by Gomez et al. [34] for traditional Mexican cheese, but lower than typically reported for unsalted cheese. Rennet curd contains inorganic compounds that may dissolve during glycolysis, further lowering water activity [35]. Furthermore, there is a strong positive and highly significant relationship between water activity (a_w_) and cheese moisture content [36]. In most cheese production processes, additional steps, such as salting or aging, reduce the moisture content and affect water activity. However, since our study focused on drained cheese curds without these additional treatments, the samples of retained a substantial amount of moisture and indeed exhibited different characteristics, including water activity (Table 2), compared to traditionally processed cheese.

## Data Availability

The data presented in this study are available on request from the corresponding author. The data are not publicly available due to data designed for other ongoing research should be protected before formal publication.

## Supplementary Materials

The following supporting information can be downloaded at: https://www.mdpi.com/article/10.3390/foods13040616/s1, Table S1: 3way ANOVA results for physicochemical characteristics of OLE supplemented cheese curds; Table S2: 3way ANOVA results for textural parameters of OLE supplemented cheese curds; Table S3: 3way ANOVA results for TPs, TFs, and AA in cheese curds.
