# Supplementary material for: Production of Acid and Rennet-Coagulated Cheese Enriched by Olive (Olea europaea L.) Leaf Extract—Determining the Optimal Point of Supplementation and Its Effects on Curd Characteristics
Source: Foods. 2024 Feb 18;13(4):616. doi: 10.3390/foods13040616 (PMC10887763; doi:10.3390/foods13040616)
Supplement: Supplementary file 1 [file foods-13-00616-s001.zip › Table S1.pdf]

Table S1. 3way ANOVA results for physicochemical characteristics of OLE supplemented cheese curds.

**Source of Variation**	**pH**	**% LA**	**Aw**	**Fat (%)**	**Moisture (%)**	**Protein (%)**	**SFA[*] (%)**	**FDM[**] (%)**	**Total Solids (%)**	**ΔE***
Confidence level 0.05 (95% confidence interval)	p value									
Effective concentration of OLE	0.0050	<0.0001	0.0058	<0.0001	<0.0001	<0.0001	<0.0001	0.0001	<0.0001	<0.0001
Coagulation method	<0.0001	<0.0001	0.6646	<0.0001	<0.0001	<0.0001	<0.0001	<0.0001	<0.0001	0.7783
Moment of OLE addition	<0.0001	<0.0001	<0.0001	0.0001	0.0012	0.0564	0.0063	<0.0001	0.0012	0.0024
Effective concentration of OLE x Coagulation method	0.0004	<0.0001	0.0132	0.2868	0.6071	0.7443	0.0002	0.0660	0.6071	0.2157
Effective concentration of OLE x Moment of OLE addition	<0.0001	<0.0001	0.0027	0.0373	0.1040	0.3971	0.0020	0.0016	0.1040	0.0016
Coagulation method x Moment of OLE addition	<0.0001	<0.0001	0.5170	0.0973	0.9291	0.0078	0.1948	<0.0001	0.9291	0.0034
Effective concentration of OLE x Coagulation method x Moment of OLE addition	0.0087	<0.0001	0.0162	0.0312	0.0476	0.0070	0.1324	0.0012	0.0476	<0.0001

[*]SFA (Satturated Fatty Acids)
[**]FDM (Fat in Dry Matter)
