# Supplementary material for: Production of Acid and Rennet-Coagulated Cheese Enriched by Olive (Olea europaea L.) Leaf Extract—Determining the Optimal Point of Supplementation and Its Effects on Curd Characteristics
Source: Foods. 2024 Feb 18;13(4):616. doi: 10.3390/foods13040616 (PMC10887763; doi:10.3390/foods13040616)
Supplement: Supplementary file 1 [file foods-13-00616-s001.zip › Table S2.pdf]

Table S2. 3way ANOVA results for textural parameters of OLE supplemented cheese curds.

**Source of Variation**	**Hardness**	**Gumminess**	**Chewiness**	**Springiness**
Confidence level 0.05 (95% confidence interval)	p value			
Effective concentration of OLE	0.1595	0.7266	0.0973	0.2684
Coagulation method	<0.0001	<0.0001	0.0064	<0.0001
Moment of OLE addition	<0.0001	<0.0001	<0.0001	0.1003
Effective concentration of OLE x Coagulation method	0.1148	0.0348	0.0099	0.0872
Effective concentration of OLE x Moment of OLE addition	0.6362	0.9929	0.8133	0.1966
Coagulation method x Moment of OLE addition	<0.0001	<0.0001	0.0002	0.0255
Effective concentration of OLE x Coagulation method x Moment of OLE addition	0.1519	0.1653	0.2121	0.0295
