# Supplementary material for: Production of Acid and Rennet-Coagulated Cheese Enriched by Olive (Olea europaea L.) Leaf Extract—Determining the Optimal Point of Supplementation and Its Effects on Curd Characteristics
Source: Foods. 2024 Feb 18;13(4):616. doi: 10.3390/foods13040616 (PMC10887763; doi:10.3390/foods13040616)
Supplement: Supplementary file 1 [file foods-13-00616-s001.zip › Table S3.pdf]

Table S3. 3way ANOVA results for TPs, TFs and AA in cheese curds.

**Source of Variation**	**Total Phenols**	**Total flavonoids**	**Antioxidant activity**
Confidence level 0.05 (95% confidence interval)		p value	
Effective concentration of OLE	<0.0001	<0.0001	<0.0001
Coagulation method	0.3003	<0.0001	<0.0001
Moment of OLE addition	0.0006	<0.0001	<0.0001
Effective concentration of OLE x Coagulation method	<0.0001	<0.0001	<0.0001
Effective concentration of OLE x Moment of OLE addition	<0.0001	<0.0001	<0.0001
Coagulation method x Moment of OLE addition	<0.0001	<0.0001	<0.0001
Effective concentration of OLE x Coagulation method x Moment of OLE addition	<0.0001	<0.0001	<0.0001
